# Incidence of Late-Diagnosed Hip Dislocation After Universal Clinical Screening in Sweden

**DOI:** 10.1001/jamanetworkopen.2019.14779

**Published:** 2019-11-08

**Authors:** Daniel Wenger, Henrik Düppe, Jan-Åke Nilsson, Carl Johan Tiderius

**Affiliations:** 1 Lund University, Lund, Sweden; 2 Department of Orthopedics, Skåne University Hospital, Malmö, Sweden; 3 Department of Orthopedics, Skåne University Hospital, Lund, Sweden; 4 Department of Rheumatology, Skåne University Hospital, Lund, Sweden

## Abstract

**Importance:**

Developmental dysplasia of the hip, including late-diagnosed hip dislocation, is the leading cause of hip arthroplasties in young adults. Early treatment is essential for a good prognosis. Before the institution of a national screening program, a minimum of 0.9 per 1000 Swedish children were affected.

**Objectives:**

To evaluate the incidence of late-diagnosed hip dislocation among children who undergo clinical screening as neonates and to study antenatal and perinatal risk factors for late-diagnosed hip dislocation.

**Design, Setting, and Participants:**

This nested case-control study included children born in Sweden from January 1, 2000, through December 31, 2009. All maternity wards, maternal health units, and orthopedic departments in Sweden participated. Children with a late-diagnosed hip dislocation were matched by sex and birth year to randomly selected controls in a 1:10 ratio. Potential risk factors in pregnant women and newborns were recorded, and cases of hip dislocation were registered. Observation time ranged from 8 to 18 years, with the last data analysis in January 2019.

**Exposures:**

Clinical hip examinations neonatally and at 6 to 8 weeks, 6 months, and 10 to 12 months.

**Main Outcomes and Measures:**

Hip dislocation diagnosed more than 14 days after birth, age at diagnosis, and severity of dislocation.

**Results:**

Among 1 013 589 live births (521 728 [51.5%] boys), 126 children (0.12 [95% CI, 0.10-0.15] per 1000 live births) had a late-diagnosed hip dislocation at a median age of 31.4 weeks (interquartile range, 16.1-67.1 weeks; 95% CI, 27.4-44.1 weeks). The incidence of late-diagnosed hip dislocation was 9 times higher among girls (113 of 491 861; 0.23 [95% CI, 0.19-0.28] per 1000 live births) than among boys (13 of 521 728; 0.02 [95% CI, 0.01-0.04] per 1000 live births). Twenty-one children (0.02 per 1000 live births) had high (severe) dislocations. Breech delivery (adjusted odds ratio, 3.07; 95% CI, 1.34-7.02), short body length at birth (adjusted odds ratio, 0.86; 95% CI, 0.76-0.98, per additional 1 cm), and being large for gestational age (adjusted odds ratio, 3.59; 95% CI, 1.30-9.95) were independent risk factors. Maternal smoking at the first visit to the maternal health care center was less common among children with hip dislocation (adjusted odds ratio, 0.16; 95% CI, 0.04-0.70).

**Conclusions and Relevance:**

Compared with historical data, the incidence of late-diagnosed hip dislocation in Swedish-born children appears to have decreased substantially since the screening program was initiated, as have the age at detection and disease severity. Similar screening programs should also be possible to institute in upper-middle- and lower-middle-income countries.

## Key Points

**Question:**

What is the incidence of late-diagnosed hip dislocation among children undergoing clinical screening for hip instability as neonates?

**Findings:**

In this nested case-control study of 1 million children in a national registry in Sweden, 126 children (0.12 per 1000 live births) were diagnosed with hip dislocation later than 2 weeks after birth, of whom 21 (0.02 per 1000 live births) had high (severe) dislocations.

**Meaning:**

Compared with historical data, the incidence of late-diagnosed hip dislocation in Swedish-born children appears to have decreased substantially since the screening program was initiated, as have the age at detection and disease severity.

## Teaser

This nested case-control study investigates the incidence and risk factors of late-diagnosed hip dislocation among Swedish children undergoing clinical screening for hip instability as neonates.

## Introduction

Developmental dysplasia of the hip (DDH) is a condition that includes neonatal instability of the hip, acetabular dysplasia, and hip dislocation. An untreated hip dislocation is associated with poor function and a high risk of early-onset osteoarthritis.^1^ Developmental dysplasia of the hip is a leading cause of total hip arthroplasty in young adults.^2^

Clinical screening for neonatal instability of the hip, using the Ortolani reduction test, was gradually introduced in Sweden starting in 1950.^3^ The subluxation provocation, today known as the Barlow test, was added to the examination protocol in 1956.^3^

Before the advent of screening for neonatal instability of the hip in Sweden, the minimum incidence of late-diagnosed hip dislocation was 0.9 per 1000 live births.^4^ At the Institutes for the Disabled, one-third of the patients were admitted because of DDH.^4^ After the introduction of screening, both in maternity wards and at child health care centers, the incidence of hip dislocation decreased, as did the mean age at diagnosis.^5^

Previous studies^6,7,8,9,10,11^ on clinical screening for DDH used data collected retrospectively from single or a few centers of treatment, including birth cohorts up to approximately 100 000 children. In Germany and Austria, the incidence of DDH has decreased since universal sonographic screening programs were introduced.^12,13^ In the United Kingdom, however, selective sonographic screening does not seem to have influenced the incidence of DDH.^14^

In Sweden, almost every child is born in a hospital. All newborns undergo a mandatory clinical examination by a pediatrician, normally before discharge from the maternity ward. This examination includes the hips, and if there is suspicion of dislocation or instability, the child is referred to an orthopedic surgeon. The clinical examination is sometimes complemented by dynamic or static ultrasonographic hip examination.^15,16^ Further clinical hip examinations are done by general practitioners at child health care centers at 6 to 8 weeks, 6 months, and 10 to 12 months.

The main purpose of this study was to evaluate the incidence of late-diagnosed hip dislocation in children born in Sweden using a nationwide prospective registry of late-diagnosed hip dislocations. We hypothesized that the incidence of late-diagnosed hip dislocation would be comparatively low, both in an international perspective and compared with historical data from Sweden, but that it could possibly fluctuate over time. A secondary aim was to examine potential antenatal and perinatal risk factors for late-diagnosed hip dislocation in the same birth cohort.

## Methods

In January 2000, the Swedish Pediatric Orthopaedic Society started a prospective registry of late-diagnosed hip dislocations. All orthopedic departments in Sweden participated with designated contact persons (n = 32) who also received yearly reminders to report cases. Hospitals with subspecialized pediatric orthopedic surgeons sent yearly reports even if no cases had been found during the year. For this nested case-control study, hospital files and radiographs were collected for all children born in Sweden from January 1, 2000, through December 31, 2009, with a late-diagnosed hip dislocation, defined as later than 14 days after birth. The age of 14 days was chosen arbitrarily as inclusion criteria to the registry because the primary aim of the registry was to monitor the neonatal screening of newborns. The observation time was through December 31, 2017 (ages 8-18 years). The study was performed in accordance with the World Medical Association Declaration of Helsinki.^17^ The regional ethical review board approved the registry and the present study. Verbal informed consent was obtained from the children's parents on inclusion in the registry and they were informed about the incidence and radiograph analysis at that time. The ethical review board waived parental and child consent for the present study because the review board did not consider it necessary to ask for parental approval a second time. This study followed the Strengthening the Reporting of Observational Studies in Epidemiology (STROBE) reporting guideline.

**Figure 1.**
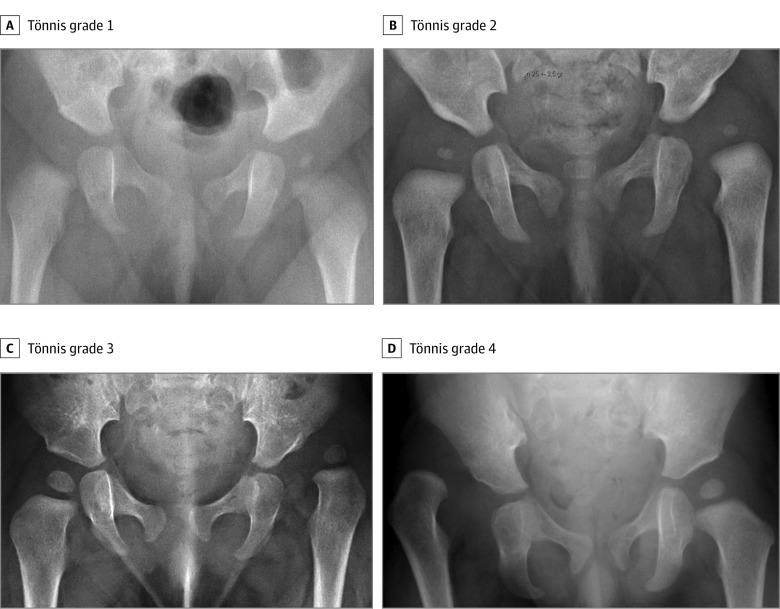
Tönnis Classification A, Grade 1: the center of the femoral head lies medial to the Perkin line (a vertical line drawn from the most lateral point of the acetabulum). Radiograph is of an infant girl with a dislocatable right hip discovered at routine examination. B, Grade 2: the center of the left femoral head lies lateral to the Perkin line. Radiograph is of an infant girl referred because of decreased hip abduction at routine examination. C, Grade 3: the center of the left femoral head lies at the level of the lateral aspect of the acetabulum. Radiograph is of an infant girl referred because of limited abduction. The mother had noted shortening of the leg. D, Grade 4: the center of the right femoral head lies above the lateral aspect of the acetabulum. Radiograph is of a male child referred because of leg length discrepancy. The parents had sought medical attention for some time before the diagnosis was made.

Neuromuscular and teratogenic dislocations and cases of isolated acetabular dysplasia with concentric stable hips were not included. Children born outside Sweden were not included. The age at diagnosis was defined by the date when an orthopedic surgeon documented the diagnosis in the hospital files. The Tönnis grade was used for describing the radiographic severity of dislocation (Figure 1).^18^ All radiographs were reviewed by the same author (D.W.). A Tönnis grade of 3 or 4 was considered to indicate a high dislocation. The number of live births in Sweden^19^ during the 10-year study period (2000-2009), stratified by year and sex, was obtained from Statistics Sweden.

For analysis of potential antenatal and perinatal risk factors for hip dislocation, data from the Medical Birth Registry were accessed. The Medical Birth Registry is run by the National Board of Health and Welfare and has prospectively registered data on pregnancies that have resulted in birth since 1973.^20^ Registration starts at the first visit to the maternal health care center, typically at gestational weeks 8 to 10, and includes the perinatal period. All maternal units in Sweden report to the Medical Birth Registry. During the study period, 98.4% of the live births registered by Statistics Sweden were registered in the Medical Birth Registry. For each child with a late-diagnosed hip dislocation, 10 control children were randomly selected, matched by sex and year of birth. The analyzed variables were (1) maternal body weight, body length, and body mass index, (2) maternal body weight and body mass index at delivery, (3) maternal age, (4) length of pregnancy, (5) chronic disease such as kidney disease, diabetes, epilepsy, asthma, inflammatory bowel disease, systemic lupus erythematosus, or hypertension, (6) smoking and snuff use, (7) delivery of a previous live birth, (8) neonate birth weight, body length, and head circumference, (9) Apgar score at 1, 5, and 10 minutes, (10) breech delivery, previous cesarean delivery, cesarean delivery, delivery by forceps, or delivery by suction bell, (11) multiple pregnancies, and (12) child being small or large for gestational age.

### Statistical Analysis

The incidence (or risk, described as cases per 1000 live births) of late-diagnosed hip dislocation among boys and girls was calculated using binomial distribution (exact method). Time trends in the annual incidence of late-diagnosed hip dislocation were analyzed using Poisson regression in a mixed-effects model. Differences in proportions with 95% CIs for categorical variables were calculated using the *z* test. Continuous variables for matched data were compared using a generalized linear model (analysis of variance). Matching sets were omitted from analysis if data were missing for a case. Maternal body mass index (calculated as weight in kilograms divided by height in meters squared) was defined as low (<20), normal (20-25), or high (>25) for analysis of a possible nonlinear association with hip dislocation risk. Odds ratios with 95% CI were calculated using conditional logistic regression analysis, with variables that yielded 2-sided *P* < .20 included. Because of collinearity between body mass index and body weight, the latter was omitted from the conditional logistic regression analysis. The median age at diagnosis with 95% CI was calculated using ratio statistics. A 2-sided α = .05 was chosen as the level of statistical significance for inferential statistics. Statistical analyses were performed with SPSS statistics, version 22.0 for Mac (IBM Corp). Last analysis of incidence and demographics was performed on January 28, 2016 (the last case in the birth cohort was diagnosed on October 17, 2011). Analysis of risk factors was performed in January 2019, at which time we could verify that no further cases had been diagnosed from January 1, 2016, through December 31, 2017.

## Results

**Figure 2.**
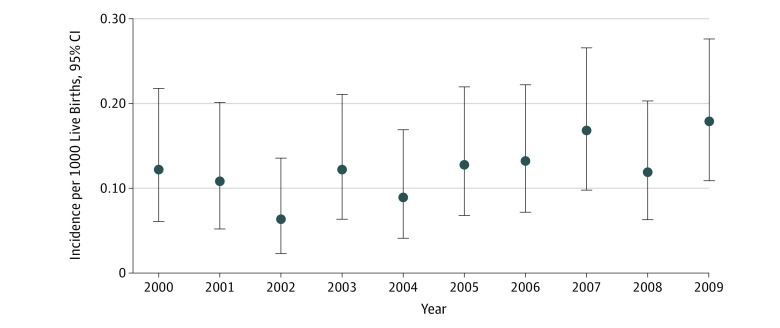
Yearly Incidence (95% CI) of Late-Diagnosed Hip Dislocation in Sweden Error bars indicate 95% CIs.

**Figure 3.**
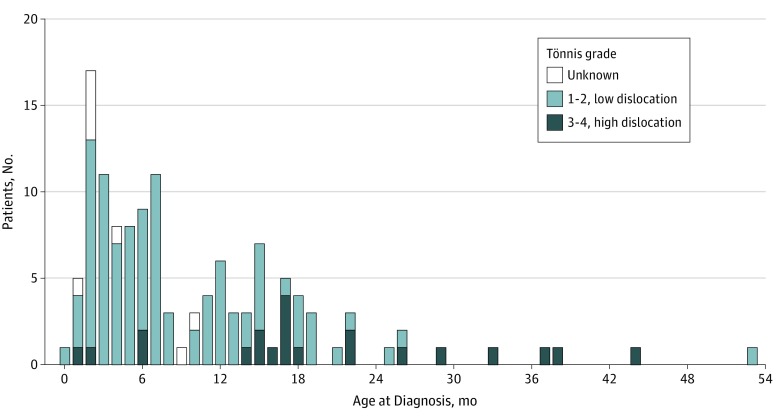
Age at Diagnosis Among 126 Children With Late-Diagnosed Hip Dislocation in Sweden In bilateral cases, the Tönnis grade of the more severely affected hip is given. Among 8 children, there was no prereduction radiograph.

There were 1 013 589 live births (491 861 [48.5%] girls and 521 728 [51.5%] boys) in Sweden during the study period. Of 126 children (0.12 [95% CI, 0.10-0.15] per 1000 live births), 113 girls (89.7%) and 13 boys (10.3%) had a late-diagnosed hip dislocation. The median age at diagnosis was 31.4 weeks (interquartile range, 16.1-67.1 weeks; 95% CI, 27.4-44.1 weeks). There was no statistically significant change in the yearly incidence during the study period (β, 0.060; 95% CI, –0.002 to 0.122; *P* = .06) (Figure 2). The incidence of late-diagnosed hip dislocation was 9 times higher among girls (113 of 491 861; 0.23 [95% CI, 0.19-0.28] per 1000 live births) compared with boys (13 of 521 728; 0.02 [95% CI, 0.01-0.04] per 1000 live births). Of these hip dislocations, 81 (64.2%) were left-sided, 30 (23.8%) were right-sided, and 15 (11.9%) were bilateral. There was no statistically significant difference in the side distribution between girls and boys. A total of 15 children (12%) were older than 18 months at diagnosis, of whom 4 children were older than 3 years (Figure 3).

There were no prereduction radiographs available for grading of 8 children: 4 children (ages 9, 10, 11, and 12 weeks) for whom diagnosis was by ultrasonography only and 4 children (ages 7, 17, 40, and 45 weeks) for whom radiographs could not be retrieved. Twenty-one children (0.02 [95% CI; 0.01-0.03] per 1000 live births) had a high dislocation: 7 (33%) were Tönnis grade 4 (6 [86%] were bilateral), and 14 (67%) were Tönnis grade 3 (1 [7%] was bilateral). Children with high dislocations were older at the time of diagnosis compared with children with Tönnis grade 1 or 2 dislocations (Table 1). Among children diagnosed from age 2 weeks to 11 months, 4 of 73 (5.5%) had high dislocations. Among children diagnosed during their second year of life, 11 of 36 (31%) had high dislocations. Of the 9 children diagnosed after 2 years of age, 6 (67%) had high dislocations.

**Table 1.** Association Among Tönnis Grade, Age at Diagnosis, and Time to Hip Reduction

Tönnis Grade	Patients, No. (Bilateral Hip Involvement, No.)	Age, Median (IQR) [95% CI], wk
1	7 (0)	16.1 (13.0-31.0) [3.6-31.1]
2	92 (9)	31.1 (17.2-57.5) [25.1-35.7]
3	14 (1)	75.5 (55.7-98.4) [27.9-98.0]
4	7 (6)	115.7 (75.3-166.0) [6.9-191.7]
All	126^a^	31.4 (16.1-67.1) [27.4-44.1]

Abbreviation: IQR, interquartile range.

^a^ Includes 8 patients for whom radiographs were not retrieved (see Methods section).

Of the 126 children with hip dislocation, 114 (90.5%) were found in the Medical Birth Registry and 1140 matched controls were allocated. Mothers of children with hip dislocation had 3.1 days (95% CI, 0.6-5.6 days) shorter length of pregnancy than mothers of control children. Children with hip dislocation measured 5.3 mm (95% CI, 0.07-0.99 mm) shorter at birth compared with controls. Maternal smoking at the first visit to maternal health care was associated with a lower risk of child hip dislocation (adjusted odds ratio, 0.16; 95% CI, 0.04-0.70). The rate of women smoking more than 9 cigarettes daily decreased from 7.9% at 3 months before the first visit to 2.5% at the first visit and 1.1% during gestational weeks 30 to 32. Breech presentation (adjusted odds ratio, 3.07; 95% CI, 1.34-7.02), short body length at birth (adjusted odds ratio, 0.86; 95% CI, 0.76-0.98, per additional 1 cm), and being large for gestational age (adjusted odds ratio, 3.59; 95% CI, 1.30-9.95) were more common among children with hip dislocation (Table 2).

**Table 2.** Analysis of Potential Antenatal and Perinatal Risk Factors for Late-Diagnosed Hip Dislocation

Risk Factor	Children, No.^a^	Children With Hip Dislocation	Control Children	Difference (95% CI)	Adjusted Odds Ratio (95% CI)^b^
Body weight, mean (SD), kg^c^	101	67.2 (12.6)	67.9 (12.6)	–0.71 (–3.3 to 1.9)	NA
Length, mean (SD), cm	105	166 (6.3)	166 (6.3)	–0.16 (–1.4 to 1.1)	NA
BMI, mean (SD), kg/cm^2^^c^	99	24.3 (4.2)	24.5 (4.2)	–0.23 (–1.1 to 0.6)	NA
Body weight at delivery, mean (SD), kg	42	84.6 (15.1)	82.4 (15.1)	2.2 (–2.6 to 7.0)	NA
BMI at delivery, mean (SD)^d^	38	30.5 (5.3)	29.7 (5.3)	0.82 (–1.0 to 2.6)	NA
Age, y	114	30.8 (5.2)	30.5 (5.2)	0.3 (–0.7 to 1.3)	NA
Pregnancy, mean (SD), d	114	275 (13.0)	278 (13.0)	–3.1 (–0.6 to –5.6)	1.03 (0.86 to 1.23)
Birth weight, mean (SD), g	114	3440 (571.8)	3459 (571.8)	–19 (–129 to 91)	NA
Body length at birth, mean (SD), cm	113	49.5 (2.4)	50.0 (2.4)	–0.53 (–0.07 to –0.99)	0.86 (0.76 to 0.98)
Head circumference, mean (SD), cm	107	34.6 (1.6)	34.7 (1.6)	–0.05 (–0.37 to 0.27)	NA
Apgar score, mean (SD), min					
1	112	8.6 (1.3)	8.7 (1.3)	–0.07 (–0.33 to 0.18)	NA
5	112	9.7 (0.9)	9.7 (0.9)	–0.02 (–0.19 to 0.15)	NA
10	112	9.9 (0.7)	9.9 (0.7)	0.03 (–0.11 to 0.17)	NA
Chronic disease, No./total No. (%)^e^	NA	15/114 (13.2)	99/1140 (8.7)	4.5 (–1.1 to 10)	1.57 (0.78 to 3.16)
Smoking, No./total No. (%)^f^					
Previous 3 mo	NA	15/108 (13.9)	181/1071 (16.9)	–3.0 (–10 to 4.4)	NA
First visit^c^	NA	3/108 (2.8)	92/1072 (8.6)	–5.8 (–11 to –0.4)	0.16 (0.04 to 0.70)
Weeks 30-32	NA	4/104 (3.8)	63/1042 (6.0)	–2.2 (–6.9 to 2.5)	NA
Snuff use, No./total No. (%)					
Previous 3 mo	NA	4/108 (3.7)	29/1071 (2.7)	1.0 (–2.3 to 4.3)	NA
First visit^c^	NA	2/108 (1.9)	16/1072 (1.5)	0.4 (–2.1 to 2.8)	NA
Weeks 30-32	NA	1/106 (0.9)	5/1051 (0.5)	0.5 (–1.0 to 1.9)	NA
Previous live birth, No./total No. (%)	NA	57/114 (50.0)	565/1140 (49.6)	0.4 (–9.2 to 10)	NA
Breech delivery, No./total No. (%)	NA	15/107 (14.0)	60/1076 (5.6)	8.4 (3.6 to 13)	3.07 (1.34 to 7.02)
Previous cesarean delivery, No./total No. (%)	NA	12/112 (10.7)	95/1122 (8.5)	2.2 (–3.2 to 7.7)	NA
Cesarean delivery, No./total No. (%)	NA	36/114 (31.6)	199/1140 (17.5)	14 (6.6 to 22)	NA
Acute or elective surgical procedure, No./total No. (%)^g^	NA	18/35 (51.4)	84/170 (49.4)	2.0 (–16 to 20)	NA
Forceps used, No./total No. (%)	NA	1/114 (0.9)	10/1140 (0.9)	0 (–1.8 to 1.8)	NA
Suction bell used, No./total No. (%)	NA	10/114 (8.8)	95/1140 (8.3)	0.4 (–4.9 to 5.8)	NA
Multiple pregnancy, No./total No. (%)	NA	4/114 (3.5)	35/1140 (3.1)	0.4 (–2.9 to 3.8)	NA
High or low BMI, No./total No. (%)^c^^,^^h^	NA	43/99 (43.4)	446/1011 (44.1)	–0.6 (–11 to 9.6)	NA
Small for gestational age, No./total No. (%)	NA	1/110 (0.9)	20/1103 (1.8)	–0.9 (–3.5 to 1.7)	NA
Large for gestational age, No./total No. (%)	NA	9/110 (8.2)	39/1103 (3.5)	4.6 (0.8 to 8.5)	3.59 (1.30 to 9.95)

Abbreviations: BMI, body mass index (calculated as weight in kilograms divided by height in meters squared); NA, not applicable.

^a^ Number of matching sets in each analysis for continuous variables.

^b^ Adjusted odds ratios including variables yielding *P* < .20 but excluding body weight (a priori) because of collinearity with BMI and cesarean delivery (in stepwise analysis) because of collinearity with breech presentation.

^c^ Measurement at first visit to maternal health care unit, typically at pregnancy weeks 8 to 10.

^d^ Calculated as weight in kilograms divided by height in meters squared.

^e^ Ongoing or previous chronic disease.

^f^ Smokers (1-9 cigarettes per day and >9 cigarettes per day pooled together) compared with nonsmokers.

^g^ Data on acute or elective surgical procedure not specified in 30 cases.

^h^ Mothers who had low (<20) or high (>25) compared with normal (20-25) BMI.

Because there was collinearity between cesarean delivery and breech position, with 67 of 75 children (89%) in breech presentation being delivered by the cesarean approach compared with 170 of 1179 children (14%) in vertex presentation, cesarean delivery was omitted from the analysis. There was a negative collinearity between maternal smoking at first visit and being large for gestational age; 1 of 48 large-for-gestational-age children (2%) had a mother who smoked, compared with 98 of 1206 children (8.1%) who were small or normal weight for gestational age. Omitting either did not significantly affect the results. A shorter body length at birth, breech delivery, and being large for gestational age were independently associated with later-diagnosed hip dislocation. Breech delivery or being large for gestational age was present among 23 of 114 children (20.2%; 95% CI, 13.2%-28.7%) with late-diagnosed hip dislocation. Maternal smoking was independently associated with a lower risk of hip dislocation (Table 2). A shorter length of pregnancy was not independently associated with hip dislocation.

## Discussion

We have prospectively registered all cases of late-diagnosed hip dislocation in Sweden from a 10-year birth cohort of 1 million children, with an observation time of 8 to 18 years. The incidence of 0.12 per 1000 live births may reflect a high efficacy of the screening at Swedish maternity wards. Before the screening program was introduced, the minimum incidence of hip dislocation among children born in Sweden was 0.9 per 1000 live births. That number may be an underestimation because it was calculated by performing an inventory of children admitted to Institutes for the Disabled for hip dislocation (n = 641) from the birth cohort (years 1936-1945).^4^ In the prescreening era, 87% of the cases were high dislocations, 95% of which were diagnosed later than 6 months (data from 266 cases from the 1950-1952 birth cohort).^5^ At present, 64% of late presenting cases are diagnosed before 1 year of age, compared with 10% before screening started.^5^ With unchanged incidence from the prescreening time, an estimated 790 children from the studied birth cohort would have presented with high hip dislocations compared with the 21 cases (0.02 per 1000 live births) that were found.

The visits at child health care centers are tax funded and offered to all Swedish neonates, infants, and children. We consider this service to be an integral part of the screening program because it may help lower the age at detection in late-diagnosed cases. Our finding that the percentage of high dislocations was 5.5% for children diagnosed in their first year, 31% when diagnosed in the second year, and 67% when diagnosed after the second year shows that hip dislocations worsened with time. A younger age at the time of hip reduction is associated with lower risk of avascular necrosis of the femoral head after treatment.^21,22,23,24^ A higher Tönnis grade is associated with failure of closed reduction.^25^ Both the age at reduction and the Tönnis grade are associated with the radiographic outcome at skeletal maturity, which is associated with osteoarthritis necessitating total hip arthroplasty.^21,26^ During long-term follow-up after open reduction (ages 1.5-5 years), 46% of individuals with the affected hips had undergone hip arthroplasty for secondary osteoarthritis by 45 years of age.^27^ In a similar cohort treated primarily with closed reduction (ages 1.5-5.2 years), 49% of individuals had osteoarthritis at 50 years of age, with 32% having a hip arthroplasty by 52 years of age.^28^

Although neonatal instability of the hip often resolves spontaneously during the first weeks of life, children who are cleared after a wait-and-see period of a few days may present later with a hip dislocation.^7^ In a randomized clinical trial that compared ultrasonography with clinical examinations for guiding treatment, 649 infants with neonatal instability of the hip were recruited up to 43 days of age. Treatment was initiated directly if an abnormal finding was confirmed but delayed to 8 weeks in uncertain cases. After this seemingly small delay, 46 infants (7.3%) needed subsequent surgery and 5 (0.8%) were not walking by 2 years of age.^29^ The best outcome may be expected if treatment is started during the neonatal period, when the risk of avascular necrosis is low.^7,30,31,32^

Two previous studies^12,13^ have evaluated universal ultrasonographic screening programs on a nationwide scale. In Germany, the rate of surgical procedures for DDH has been reduced from a historic minimum rate of 1 per 1000 live births to 0.26 per 1000 live births since the introduction of a screening program based on ultrasonographic examinations.^12^ In Austria, ultrasonographic screening has also been associated with a lowered rate of surgical procedures for DDH. However, just the rate of surgical open reductions in Austria (0.12 per 1000 live births) equals the total rate of late presenting hip dislocations in Sweden.^13^

In the United Kingdom, ultrasonographic screening of children with risk factors (selective screening) has not been associated with lower incidence of DDH diagnosed later than 1 year of age, with 1.28 per 1000 children aged 1 to 8 years affected.^14^ Our finding that only 20% of the children with hip dislocation had breech presentation or were large for gestational age may partly explain the failure of the UK screening program, in which only children with first-degree family history or breech presentation are screened.

One problem with ultrasonographic assessment of acetabular morphologic features is its weak prognostic value among newborns. With a stable, concentric hip, the acetabulum develops normally, even in cases in which the hip appears to be dysplastic during the first weeks of life. This finding was reported by Graf^16^ in his description of the widespread ultrasonographic technique that bears his name. If decentration of the femoral head did not occur, Graf considered acetabular dysplasia to be a matter of maturity for infants up to 3 months of age. Consequently, ultrasonographic screening programs typically allow for long wait-and-see periods during which many of the observed hips normalize spontaneously.^12,13,29,33^ For hips that do not normalize, a delay of 1 to 3 months may be associated with an increased risk of treatment failure and serious complications, as discussed above.^29^ Because of these findings, Swedish neonates are treated without delay and diagnosis later than 2 weeks age is considered to be late in the registry.

Clinical screening requires skilled examiners to be effective and efficient. If the examinations at the maternal ward are left to a large number of rotating junior physicians, both referral rates for treatment and the rate of late-diagnosed dislocations may increase.^8^ Therefore, one of the main aims of the Swedish registry is to continually monitor the screening program. We found no change in yearly incidence during the study period.

Because the prevalence of confounding factors, such as the practice of swaddling, the use of baby carriers with hips abducted, or positioning sleeping children prone or supine, may change in a population, conclusions cannot be made about causality from this observational study in isolation. We are not aware of any randomized clinical trial comparing clinical screening with no screening (or sham screening). The best available evidence to support the notion of a causal effect of clinical screening comes from numerous comparative cohort studies^6,8,9,10,11,31,34,35,36,37,38^ in which significant reductions in the frequency of late-diagnosed hip dislocation are reported in cohorts examined by more experienced or specifically trained staff, comparing different units^9,10,11,34,35,36,37,38^ and longitudinally at the same unit.^6,8,31^ Two randomized clinical trials assessed the efficacy of ultrasonographic screening but did not find such screening to be efficacious.^33,39^

Female sex, breech presentation, and high birth weight in a dose-dependent association have previously been defined as independent risk factors for DDH.^40^ Other reported risk factors include heredity, oligohydramniosis, and primiparity.^41^ Although we found a 14% decrease in the risk of late hip dislocation for every centimeter of birth length, we are not aware of any previous study showing an association between birth length and risk of DDH.

The finding that maternal smoking during early pregnancy was associated with a lower risk of having a child with late-diagnosed hip dislocation must be interpreted with care. Smoking during pregnancy is associated with increased risks of stillbirth, very preterm birth, and infant death during the first year of life.^42,43,44^ Of note, most women who smoked were able to quit or significantly reduce their tobacco use during pregnancy. Antenatal estrogen effects on ligamentous laxity have previously been hypothesized^45^ to contribute to the large difference in risk of neonatal instability of the hip and hip dislocation between the sexes. A case-control study of 2185 neonates reported higher levels of 17β-estradiol in cord blood samples from girls with neonatal instability of the hip compared with controls, but the opposite finding was reported among boys.^46^ Smoking has antiestrogen effects in women,^45^ and lower levels of estriol in cord blood samples from women who smoke compared with nonsmokers have been reported.^47^ Although a protective biological effect of smoking on DDH can be imagined, our finding is based on few cases and should only be viewed as hypothesis generating until confirmed or rejected in further studies.

### Strengths and Limitations

Strengths of this study include the cohort size, with 1 million children examined in the program, enabling adequate statistical power for analysis of risk factors; the prospective registration of cases and potential risk factors; 100% coverage (all hospitals treating DDH and all maternity units in Sweden); the observation time of 8 to 18 years; and the nationwide catchment area. The use of matched controls, randomly selected from the entire birth cohort, is another methodologic strength.

This study has limitations. Though we have found a marked difference by comparing 2 periods, level 1 evidence supporting screening for hip instability is still lacking. Comparisons with historical Swedish data are somewhat flawed because in the 1936-1945 birth cohort, only children admitted to Institutes for the Disabled were included^4^ and in the 1950-1952 birth cohort, early diagnosis was defined as during the first month of life.^5^ Both these differences could cause the present study to include patients who would not have been included in the historic data. There is a possibility of unreported cases among children who moved from Sweden during their first years of life. However, such missed registrations should be few because only 12% of children were older than 18 months at diagnosis. Including the entire country in the study may lead to fewer cases missed because of relocation compared with studies from single centers or regions within countries. There is also a possibility of unreported cases from the participating units or if a case was treated in a general orthopedic department without consulting a pediatric orthopedic surgeon. We believe this to be unlikely although our study lacks hospital admission data for support. Before ossification begins in the femoral head epiphysis, the Tönnis grading system may be unreliable in cases bordering between 2 grades.^48^ Despite this limitation, the Tönnis grading system is prognostic of outcome as discussed earlier.

## Conclusions

The incidence of late-diagnosed hip dislocation after clinical screening was 0.12 per 1000 live births in this cohort of 1 million children. Compared with historic data, this incidence has decreased substantially since the screening program was initiated, as have the age at detection and disease severity. Considering previous evidence, the screening program may explain a portion of these improvements. Similar screening programs should also be possible to institute in upper-middle- and lower-middle-income countries.
